# Surgical management of peripheral nerve sheath tumours: a 10-year retrospective UK study

**DOI:** 10.1016/j.bas.2026.106171

**Published:** 2026-07-15

**Authors:** Rahul Shah, Tamara Tajsic, Sruthi Ranganathan, Rikin Trivedi

**Affiliations:** a University of Cambridge School of Clinical Medicine, Cambridge, United Kingdom; b Department of Neurosurgery, Cambridge University Hospitals NHS Trust, Cambridge, United Kingdom

**Keywords:** Peripheral nerve sheath tumours, Complications, Retrospective study, Predictors

## Abstract

**Introduction:**

Peripheral nerve sheath tumours (PNSTs) are predominantly benign lesions. Surgical resection is the gold-standard treatment when symptoms are troublesome, progressive and/or when formal tissue diagnosis is necessary; however, the risk of postoperative neurological deficits and the predictors of outcome are not well defined.

**Research question:**

What are the clinical characteristics, postoperative outcomes, and predictors of complications following PNST surgery?

**Material and methods:**

A retrospective chart review was conducted of all consecutive PNST resections performed at a single tertiary NHS neurosurgical centre over a ten-year period between 1st March 2015 and 31st May 2025. Demographic data, presenting symptoms, tumour characteristics and follow-up outcomes were collected and analysed. Binomial logistic regression was used to identify predictors of worse sensory outcomes.

**Results:**

83 surgeries were performed in 77 patients (median age 48 years, 50.6% male). 74.7% of tumours were schwannoma. Pain was the predominant presenting symptom (in 78.3%), and improved postoperatively in 93.8% of cases, with no new postoperative pain. New functionally impairing postoperative sensory symptoms or weakness occurred in 2.5% and 1.3% of patients, respectively. Preoperative neuropathic tenderness was independently associated with worse sensory outcome.

**Discussion and conclusion:**

PNST surgery offers reliable pain relief with a low risk of severe neurological morbidity. Sensory disturbances are common but usually mild. Patients with preoperative nerve irritability on examination may be at higher risk of postoperative sensory deficits and should be appropriately counselled.

## Highlights

• Peripheral nerve sheath tumours (PNSTs) might require surgical management.
• There is heterogeneity in the postoperative outcomes after surgery for PNSTs.
• Predictors of outcomes post-surgery for PSNTs are poorly defined.
• There is a low risk of severe postoperative sensory deficits and motor weakness.
• Age below 50, positive Tinel's sign are some predictors of poor outcomes.

## 1 Introduction

Peripheral nerve tumours (PNTs) are neuroectodermal in origin (Ariel, 1983). Peripheral nerve sheath tumours (PNSTs) represent the most common subset of PNTs, and are predominantly benign in nature (Ariel, 1983). The most frequently encountered are solitary schwannoma, which have an incidence of approximately 0.6 per 100,000 people annually (Hanemann and Evans, 2006), and neurofibroma, which is typically associated with neurocutaneous syndromes such as neurofibromatosis type 1 or 2 and schwannomatosis. Malignant PNSTs are rare (they make up 5-10% of all PNSTs (Gosk et al., 2015)), but are particularly aggressive with unfavourable prognoses (Kim et al., 2005).

PNSTs can rarely be associated with radiation or trauma, but most commonly arise sporadically from peripheral nerves in the neck and limbs (Öhlén et al., 2025). They occur most often in the third to sixth decade of life (Ogose et al., 1999). No association has been demonstrated with gender or ethnicity (Öhlén et al., 2025).

Clinically, benign PNSTs most often present with a palpable mass, pain and paraesthesia due to compression of the nerve. Motor deficits are less frequent (Hirai et al., 2019; Granlund et al., 2021). MRI is the typical imaging modality used to characterise the tumour and plan treatment, and there is a limited role for fine needle biopsy and electrophysiological studies pre-operatively (Öhlén et al., 2025; Desai, 2017). Surgical resection is the gold-standard treatment. Given schwannomas arise from a single nerve fascicle and are well-encapsulated, enucleation of the tumour with minimal damage to the nerve is often achievable. Neurofibromas may involve several nerve fascicles and have a more infiltrative expansion pattern, which can make surgery more challenging (Levi et al., 2010).

Surgical resection with the goal of preserving neurological function is not without risk, so it is generally reserved for when: 1) symptoms are especially troublesome, 2) when they are likely to get worse without intervention, and/or 3) to histologically rule out malignant PNST. Sensory deficits are the most common post-operative complication; other unfavourable complications include new or worsened weakness or long-term pain (Gosk et al., 2015; Granlund et al., 2021; Levi et al., 2010).

Despite several large retrospective series, predictors of postoperative outcome and complication risk remain incompletely defined. This study aims to review a single-centre experience with PNST surgery over 10 years, focusing on clinical presentation, outcomes and predictors of complications.

## 2 Methods

A retrospective chart review of all, consecutive peripheral nerve surgeries at a single tertiary NHS neurosurgical centre from 1st March 2015 to 31st May 2025 was conducted. Cases were identified via the electronic patient record system's operation log. Surgeries undertaken on imaging-confirmed peripheral nerve tumours on patients of any age were included.

In total, 83 surgeries were included.

We collected the following data: demographic patient characteristics including the presence of known neurocutaneous conditions, presenting symptoms and examination findings, symptom duration, lesion characteristics such as anatomical location, diameter in the longest axis and histology, and follow-up outcome.

Pre-operative diagnosis was made using MRI imaging for all cases and biopsy was performed for 7 out of 83 tumours. Tumour size was taken from radiology reports or intra-operative measurements; in 9 cases this was limited when imaging records were held by different hospital trusts and so were not available for retrospective analysis.

All surgeries were performed by the same senior surgeon. For all brachial plexus, motor or mixed nerves, we used continuous EMG monitoring and direct nerve stimulation. Intra-operative ultrasound was not used for cases included in this study. Extent of tumour resection was assessed by macroscopic inspection at surgery.

Follow-up is routinely performed approximately 3-4 months post-operatively at our institution. In this series, median time to first follow-up was 3.6 months (range 0.8-12.0 months). Longer term follow-up was left open for patients to access as and when necessary.

Outcomes were reported in the symptom specific domains of pain, weakness and sensory symptoms. The development of new post-operative symptoms was specifically noted. The motor component was measured using the MRC scale for muscle strength (0-5). Qualitative assessment of muscle wasting performed by comparison with the unaffected limb, or patient questioning. Quantitative pain severity was not routinely recorded in all cases, but was always compared pre-/post-op, from patient questioning.

### 2.1 Statistical analysis

Median, standard deviation and minimum/maximum ranges were used to describe continuous variables. Numbers and percentages were used to describe categorial variables. A binomial logistic regression analysis was done to identify the predictors of new numbness or paraesthesia or worsening of a preoperative sensory deficit post-operatively.

Given no patients reported pain as a new complication and only 3 patients had worsening of their preoperative pain, a pain outcome was not included as a dependent variable for the regression analysis. Similarly, given new severe weakness resistant to improvement with physiotherapy was not noted, and only one patient had worsening of pre-existing weakness, a weakness outcome was not included either. Predictors used in both univariate and multivariate analysis were: age (<50), gender, tumour location: brachial plexus roots-cords vs other, positive Tinel's/neuropathic tenderness, palpable lump, major vs minor nerve, tumour size (mm), schwannoma vs other histology, and underlying confirmed/suspected neurocutaneous syndrome.

These variables were chosen based on a review of existing literature suggesting them as potential influencers on outcome (Öhlén et al., 2025; Hirai et al., 2019; Granlund et al., 2021; Levi et al., 2010; Siqueira et al., 2013), reinforced by clinical rationale suggesting them as relevant data for patient counselling and consent discussions that are available preoperatively.

Listwise deletion was used to manage missing values. Statistical significance was set to p < 0.05. Statistical analysis was carried out in *jamovi (Version 2.6)* (jamovi).

## 3 Results

Between 1st March 2015 and 31st May 2025, 83 peripheral nerve tumour resections were performed in 77 patients. Most patients underwent a single operation, although 4 patients required 2 procedures and 1 patient underwent 3 procedures during the study period.

### 3.1 Demographic characteristics

**Fig. 1:**
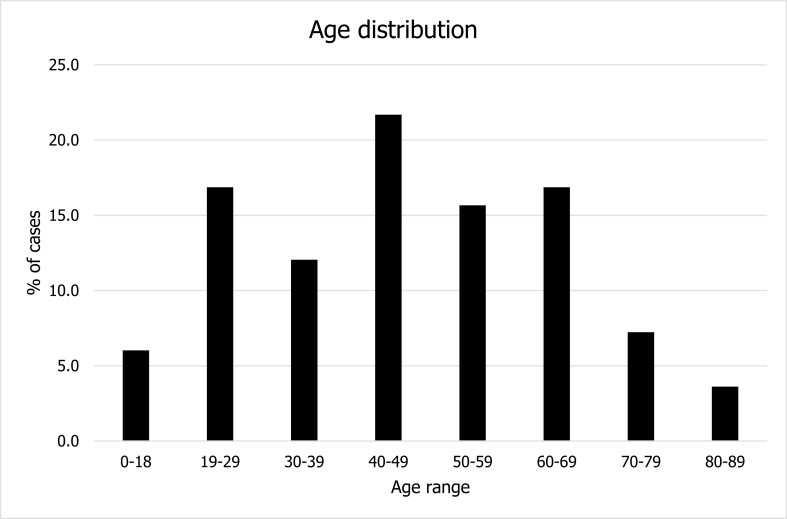
Age distribution of cases.

The cohort was evenly distributed by gender, with 51% male patients. The median age at surgery was 48 years (range 12–83 years). Patients were most commonly in the fifth decade of life (21.7%), with relatively few paediatric or very elderly cases (Fig. 1).

**Fig. 2:**
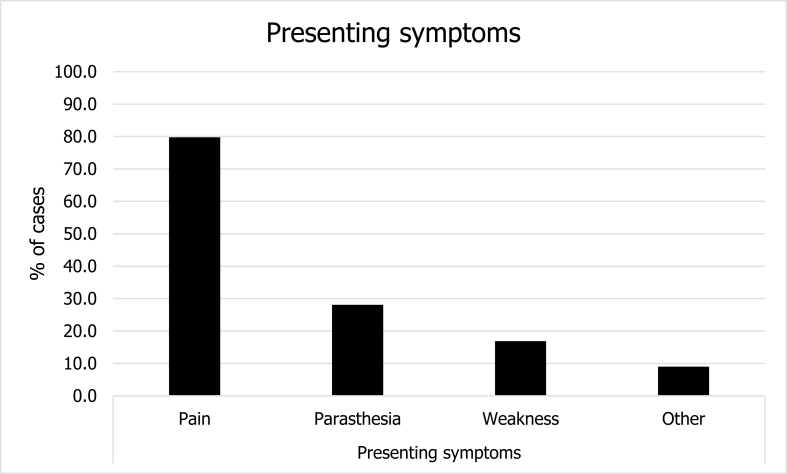
Presenting symptoms.

**Fig. 3:**
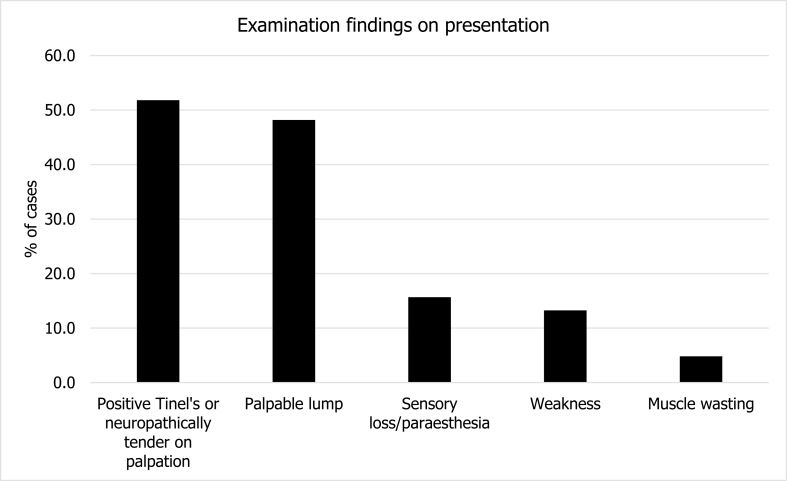
Examination findings on presentation.

Presenting symptoms were: pain in 78.3% (n = 65/83), paraesthesia in 26.5% (n = 22), and weakness in 15.7 (n = 13). Other presentations were uncommon and included gait disturbance, muscle wasting, and isolated atypical symptoms (Fig. 2). Symptoms were often longstanding at presentation, with a median duration of 24 months**,** although considerable variability was observed (SD 45.7 months). Symptom duration data were unavailable for 36 cases as they were not noted on clinic letters. On examination, positive Tinel's sign or neuropathic tenderness was present in 51.8% (n = 43)**,** a palpable lump in 48.2% (n = 40), sensory loss/paraesthesia in 15.7% (n = 13), weakness in 13.3% (n = 11), and muscle wasting in 4.8% (n = 4) (Fig. 3).

### 3.2 Lesion characteristics

The median tumour longest-axis diameter was 28 mm (SD 16.4), with tumour sizes ranging from 7 mm to 87 mm. Lesions were located in the upper limbs in 46 cases (55.4%), and in the lower limbs in 33 cases (39.8%). Two cases (2.4%) involved lesions affecting more than one limb. Single cases involved the torso (1.2%) and the extracranial hypoglossal nerve (1.2%).

**Fig. 4:**
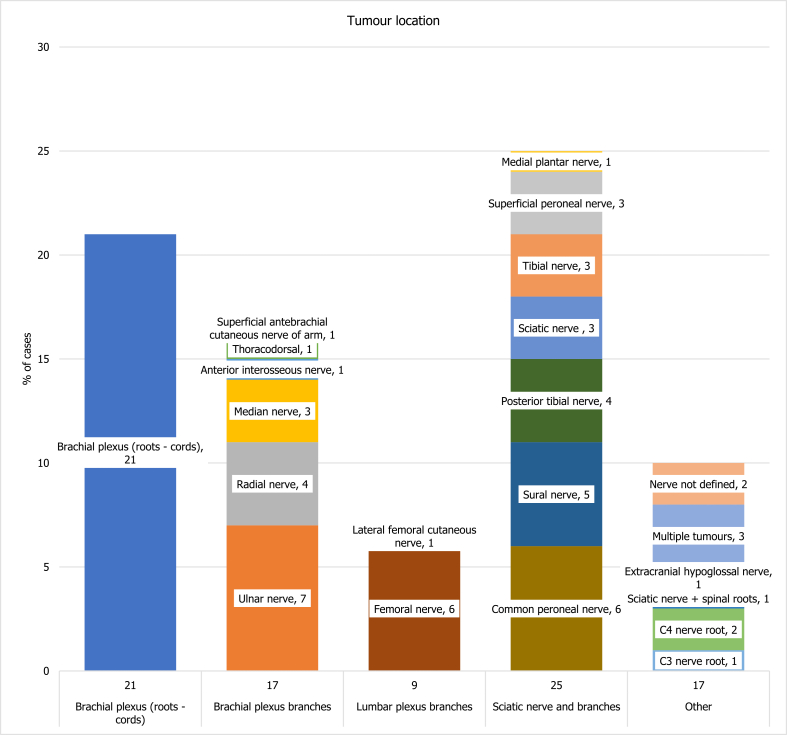
Anatomical breakdown of nerves affected by lesion.

When grouped anatomically, tumours most frequently involved the sciatic nerve and its branches (25 cases, 30.1%), followed by the brachial plexus roots, trunks, divisions and cords (21 cases, 25.3%), brachial plexus branches (17 cases, 20.5%), lumbar plexus branches (9 cases, 10.8%). A detailed breakdown of anatomical location and specific nerves affected is given in Fig. 4.

### 3.3 Histology and underlying conditions

In 95.1% of cases, the tumour was completely resected. In all cases a sample of tissue was sent for histological analysis. This most commonly demonstrated schwannoma, accounting for 62 cases (74.7%). Neurofibroma was identified in 4 cases (4.8%). Lipoma, intraneural ganglion cyst, and traumatic neuroma were each identified in 2 cases (2.4%). Only 1 malignant tumour, a synovial sarcoma, was found (1.1%).

NF2 was present in 10 patients (13.0%). Schwannomatosis with a confirmed genetic diagnosis was present in 3 patients (3.9%), schwannomatosis without formal genetic confirmation in 2 patients (2.6%), NF1 in 1 patient (1.3%), and genetic investigations were ongoing in 1 patient (1.3%).

### 3.4 Outcomes

In 80/83 cases (96.4%), patients were followed up by neurosurgery at 3-4 months post-operatively. In 3 cases (3.4%), patients had follow-up with other specialties. (e.g. oncology, neurology).

In 78.8% (n = 63) of the cases, patients reported resolution of the troublesome preoperative symptoms.

Pain was present preoperatively in 65 cases (78.3%). Pain improved following surgery in 61 cases (93.8%), remained unchanged in 1 case (1.5%), and worsened in 3 cases (4.6%). No patients developed new postoperative pain.

Twenty-two cases (26.5%) presented with sensory symptoms preoperatively. At follow-up, sensory symptoms improved in 11 cases (50%), remained stable in 10 cases (45.5%), and worsened in 1 case (4.5%). New postoperative sensory symptoms occurred in 18 cases (22.5%), of which 2 (2.5%) were subjectively described as severe or significant by patients and the neurosurgeon conducting follow-up, as they were causing day-to-day functional limitations.

Preoperative weakness was present in 13 cases (16.9%). Weakness improved postoperatively in 9 cases (69.2%), remained unchanged in 3 cases (23.1%), and worsened in 1 case (7.7%). New subjective postoperative weakness occurred in 9 cases (11.3%). In 3 of these cases (3.8%), the weakness persisted at last follow-up, but in only 1 case (1.3%) was significant and affected activities of daily living. In the other 6 cases (7.5%), weakness had either significantly improved or fully resolved by last follow-up.

**Fig. 5:**
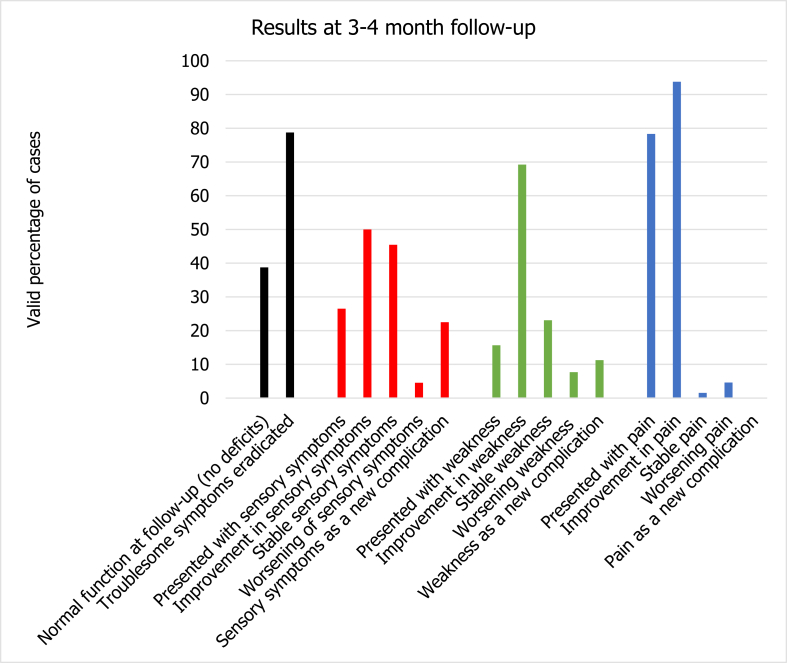
Outcomes at 3-4 months follow-up, categorised by symptom.

This data is summarised in Fig. 5.

### 3.5 Neurocutaneous syndromes

Of the 77 patients reviewed, 17 had confirmed or suspected neurocutaneous syndromes, accounting for 22 tumour resections**,** while the remaining 60 non-syndromic patients accounted for 61 resections**.**

Patients with confirmed or suspected neurocutaneous syndromes demonstrated less predictable improvement of pre-operative pain. Pain was present pre-operatively prior to 14/22 syndromic resections and 51/61 non-syndromic resections**.** Pain improved or remained stable following 12/14 (85.7%) operations in the syndromic group, compared with 50/51 (98.0%) operations in the non-syndromic group**.**

Sensory improvement or stability were similar following resections in the syndromic vs non-syndromic groups (100% vs 94.7%). Pre-operative weakness was recorded prior to 6 resections for syndromic patients and 7 resections for non-syndromic patients. Weakness improved or remained stable at first follow-up after all these resections except for 1, which was for a syndromic patient.

Following resections in syndromic vs non-syndromic patients, the incidences of new sensory deficits (18.2% vs 23.0% respectively) and new transient motor deficits (13.6% vs 9.8% respectively) were similar.

### 3.6 Predictors of complications

Predictors of new postoperative sensory loss of worsening of preoperative sensory loss included: age above 50 and major nerve location on multivariate analysis alone (p = 0.01 and p = −0.044 respectively), and the presence of a positive Tinel's sign or focal neuropathic tenderness on both univariate (p = 0.03) and multivariate analysis (p = 0.018) (Table 1).

**Table 1:** – Predictors of new postoperative sensory loss of worsening of preoperative sensory loss.

Characteristic	Reference	Univariate	Multivariate
		p-Value	p-Value
**Age below 50**	No	0.056	0.01
**Gender**	Female	0.99	0.573
**Brachial plexus roots-cords vs other location**	Other location	0.557	0.824
**Positive Tinel's sign/tenderness on palpation**	No	0.03	0.018
**Palpable lump**	No	0.155	0.281
**Major/minor nerve**	Minor	0.242	0.044
**Tumour size (longest axis, mm)**	N/A	0.297	0.062
**Schwannoma vs other histology**	Other histology	0.178	0.185
**Underlying confirmed or suspected neurocutaneous syndrome**	No	0.557	0.169

## 4 Discussion

This retrospective study analysed the demographic characteristics, lesion characteristics and postoperative outcome for 77 patients with 83 PNSTs. The goals of surgery, i.e. acquiring of tissue samples for histological diagnosis, symptom relief and prevention of symptom progression, were achieved in most cases.

### 4.1 Comparison of preoperative data with other cohorts

The age and gender profile in our cohort (median 48 years, 50.6% male) is similar to other studies, with no strong gender predilection (Hirai et al., 2019; Granlund et al., 2021; Levi et al., 2010). In contrast to extremity-only schwannoma studies our case-mix includes plexus and root-level lesions. This resembles large studies such as Kim et al.‘s 397-case series (Kim et al., 2005), and Desai's 442-case series that had substantial brachial plexus components (Desai, 2017). Schwannoma was the dominant tumour subtype in our study, as in most mixed-PNST cohorts (Öhlén et al., 2025; Ogose et al., 1999; Carvajal et al., 2011), but compared to other large studies by Levi et al. and Kim et al., our neurofibroma proportion was lower (4.8%) (Kim et al., 2005; Levi et al., 2010). Direct tumour size comparisons across studies are constrained by heterogenous reporting (diameter vs volume). Our median longest-axis diameter (28 mm) is close to Granlund et al. (median size 24 mm) and is within the range of typical extremity only schwannoma series (Hirai et al., 2019; Granlund et al., 2021). Objective signs in our cohort, particularly positive Tinel's/neuropathic tenderness and palpable lump, were present in approximately half of cases. This is lower than in some older surgical series that report very high rates of positive Tinel and paraesthesia, such as Gosk et al. (positive Hoffmann–Tinel 95.6%, paraesthesia 93.4%, palpable tumour mass 100%) (Gosk et al., 2015).

### 4.2 Symptom outcomes

Overall, nearly 80% of patients reported no troublesome symptoms at 3-4 months after surgery.

Pain was the dominant presenting symptom in our cohort (78.3%) and showed the most consistent postoperative improvement (over 90% of patients’ pain improved, with no new cases of postoperative pain). These findings align closely with other studies: Öhlén et al. reported pain improvement in 94% of patients, El Sayed et al. in 89.6% and Granlund et al. in 76% for local pain and 97% for radiating pain (Öhlén et al., 2025; Granlund et al., 2021; El Sayed et al., 2022). This consistency of pain relief following surgery across studies, supports pain as the most reliable indication for surgery and the most likely symptom to improve postoperatively.

Preoperative weakness was present in 15.7% of cases in our series, comparable to the 16% reported by Öhlén et al. but higher than the 3–6% reported in schwannoma-only extremity cohorts such as El Sayed et al. (3.3%) and Hirai et al. (4.2%) (Öhlén et al., 2025; Granlund et al., 2021; El Sayed et al., 2022). Postoperatively, weakness improved or was stable in 92.3% of patients in whom it was present pre-operatively at last follow-up. Transient new postoperative weakness occurred in 9 cases (11.3%), which had either resolved or improved at last follow-up; but persistent severe, functionally impairing weakness, was evident in only a very small minority (1.3%). This is of a similar magnitude to other series, including El Sayed et al. (4.6%) and Hirai (5.67%), with the differences likely to be accounted for by the heterogeneity grading of the weakness (Hirai et al., 2019; El Sayed et al., 2022). Hirai et al. confirm that at final follow-up, which ranged from 1 to 96 months, the MRC grade for the weakness in question was ≤3 in 4 cases (i.e. approximately 3%) (Hirai et al., 2019). El Sayed et al. did not provide detail on the extent of the new postoperative weakness in their cohort, who were followed up for an average of 5 months (El Sayed et al., 2022).

In our cohort, of the 22% of patients who presented with sensory symptoms, over 95% had their symptoms improve or stay stable. New sensory symptoms occurred in 22.5% of patients, but were only severe or functionally impairing in 2.5%, and attributable to paraesthesia rather than numbness or dysaethesiae. This is similar to Öhlén et al., who reported new postoperative sensory deficits in 35% of patients, and Hirai et al., in 29.8% of cases (Öhlén et al., 2025; Hirai et al., 2019). Rates are lower in series by El Sayed et al. and Granlund et al., but crucially, they exclusively analysed extremity schwannomas (Granlund et al., 2021; El Sayed et al., 2022); our mixed cohort rates of sensory complications are higher due to contribution from plexus and nerve root tumours. Furthermore, there is heterogeneity in reporting and sizeable variation in the reported incidence (Levi et al., 2010; Siqueira et al., 2013; Carvajal et al., 2011); Granlund et al. focus their analysis only on ‘significant’ symptoms at follow-up (Granlund et al., 2021). Sensory loss following PNST surgery can be considered an expected complication, for a number of reasons, and follow-up of 3-4 months does not allow determination of whether these are always permanent, but from anecdotal longitudinal experience at our regional centre, late re-presentation is very rare, and only 2 patients in this series (2.5%) had paraesthesia that was described as severe.

In patients with confirmed or suspected neurocutaneous syndromes, pain improved or stayed stable in a slightly lower proportion of cases (85.7% vs 98.0%) compared to non-syndromic patients. However, the improvement in sensory symptoms and weakness, and incidence of new deficits, were largely equivalent. An important caveat is that the burden of disease for a syndromic patient may lead them to report less concern for minor post-surgical deficits compared to patients with isolated PNSTs, skewing the results (Hébert-et al., 2015). However, our results add weight to the conclusion, that despite co-existing neuropathology, surgery should still be considered for syndromic patients with the appropriate counselling (Bendon et al., 2015).

### 4.3 Predictors of poor outcomes

Younger age emerged as an predictor of new sensory deficit or worsening of preoperative deficit in multivariate (but not univariate analysis), closely mirroring similar results from Öhlén et al. who reported younger age as a significant risk factor for persistent or increased sensory deficits (p = 0.002) (Öhlén et al., 2025), and from Siqueira et al., who found patients under 50 years had a higher complication rate (15.2% overall; p = 0.02) (Siqueira et al., 2013). The reasoning behind this is unclear and requires further characterisation; it may be related to the higher rate of neurofibromas that arise in younger cohorts that skew outcomes. However, Hirai et al. found older age to be associated with worse sensory complications in their univariate analysis, though this similarly did not persist in multivariate analysis (Hirai et al., 2019).

Resection of tumours impinging upon ‘major’ nerves was also a predictor of new sensory deficit or worsening of preoperative deficit in multivariate but not univariate analysis. This resembles Hirai et al.‘s findings: they found the overall postoperative complication rate was 34.8%, with sensory disturbance reported in 29.8%, and major motor nerve involvement emerged as the key independent predictor of complications (p = 0.03) (Hirai et al., 2019).

A positive Tinel's sign or neuropathic tenderness was independently associated with poor sensory outcome in our cohort, both in univariate and multivariate analysis. While often reported descriptively, this variable has rarely been analysed in predictive models. El Sayed et al. reported pseudo-Tinel positivity in 55.7% of cases, and Gosk et al. reported Hoffmann–Tinel positivity in 95.6%, but neither study examined its predictive value (Gosk et al., 2015; El Sayed et al., 2022). Our findings suggest that preoperative nerve irritability may be an under-recognised predictor of poor postoperative sensory outcomes.

### 4.4 Study strengths and limitations

This study is retrospective by design, and reliance on old documentation including clinic letters precludes the quantity and quality of clinical data available for analysis. Interpreting old documentation, which often does not contain objective assessments of patient symptoms or signs, is inherently subject to bias. However, the study has a relatively large sample size, includes patients with multiple tumours and with neurocutaneous syndromes, does not restrict to one histological subtype of PNTs and carries out a regression analysis to offer relevant insights for day-to-day clinical practise.

## 5 Conclusions

When viewed in the context of existing literature, our results reinforce that surgery for peripheral nerve tumours reliably improves pain. The risk of severe postoperative sensory deficits and motor weakness as new complications is low, and severe neurological morbidity observed in this cohort is comparable to that reported in other broad benign PNST series. The identification of preoperative neuropathic tenderness as a predictor of poor sensory outcome provides clinically actionable information for preoperative counselling and shared decision-making.

## Declaration of competing interest

The authors declare that they have no known competing financial interests or personal relationships that could have appeared to influence the work reported in this paper.

## References

[bib1] Ariel I.M. (1983). Tumors of the peripheral nervous system. CA Cancer J. Clin..

[bib2] Bendon C.L., Furniss D., Giele H.P. (2015). Comparison of outcomes of peripheral nerve schwannoma excision in neurofibromatosis type 2 patients and non-neurofibromatosis type 2 patients: a case control study. J. Plast. Reconstr. Aesthetic Surg..

[bib3] Carvajal J.A., Cuartas E., Qadir R., Levi A.D., Temple H.T. (2011). Peripheral nerve sheath tumors of the foot and ankle. Foot Ankle Int..

[bib4] Desai K.I. (2017). The surgical management of symptomatic benign peripheral nerve sheath tumors of the neck and extremities: an experience of 442 cases. Neurosurgery.

[bib5] El Sayed L., H Masmejean E., Lavollé A., Biau D., Peyre M. (2022). Clinical results after surgical resection of benign solitary schwannomas: a review of 150 cases. Orthop Traumatol Surg Res.

[bib6] Gosk J., Gutkowska O., Mazurek P., Koszewicz M., Ziółkowski P. (2015). Peripheral nerve tumours: 30-year experience in the surgical treatment. Neurosurg. Rev..

[bib7] Granlund A.S., Sørensen M.S., Jensen C.L., Bech B.H., Petersen M.M. (2021). Clinical outcome after surgery on schwannomas in the extremities. World J. Orthoped..

[bib8] Hanemann C.O., Evans D.G. (2006). News on the genetics, epidemiology, medical care and translational research of Schwannomas. J. Neurol..

[bib9] Hébert-Blouin M.N., Spinner R.J. (2015). Commentary on: ‘comparison of outcomes of peripheral nerve schwannoma excision in neurofibromatosis type 2 patients and non-neurofibromatosis type 2 patients: a case control study.’. J. Plast. Reconstr. Aesthetic Surg..

[bib10] Hirai T., Kobayashi H., Akiyama T. (2019). Predictive factors for complications after surgical treatment for schwannomas of the extremities. BMC Muscoskelet. Disord..

[bib15] Öhlén E., El-Hajj V.G., Fletcher-Sandersjöö A., Edström E., Elmi-Terander A. (2025). Clinical course and predictors of outcome following surgical treatment of benign peripheral nerve sheath tumors, a single center retrospective study. Int. J. Neurosci..

[bib11] jamovi. The Jamovi Project. www.jamovi.org.

[bib12] Kim D.H., Murovic J.A., Tiel R.L., Moes G., Kline D.G. (2005). A series of 397 peripheral neural sheath tumors: 30-year experience at Louisiana State University Health Sciences Center. J. Neurosurg..

[bib13] Levi A.D., Ross A.L., Cuartas E., Qadir R., Temple H.T. (2010). The surgical management of symptomatic peripheral nerve sheath tumors. Neurosurgery.

[bib14] Ogose A., Hotta T., Morita T. (1999). Tumors of peripheral nerves: correlation of symptoms, clinical signs, imaging features, and histologic diagnosis. Skelet. Radiol..

[bib16] Siqueira M.G., Socolovsky M., Martins R.S. (2013). Surgical treatment of typical peripheral schwannomas: the risk of new postoperative deficits. Acta Neurochir (Wien).

